# Towards effective participation of the private health sector in Ghana’s COVID-19 response

**DOI:** 10.11604/pamj.supp.2020.35.2.23387

**Published:** 2020-05-22

**Authors:** Belinda Afriyie Nimako, Frank Baiden, John Koku Awoonor-Williams

**Affiliations:** 1 Ghana Health Service, Policy Planning Monitoring and Evaluation Division, Ghana; 2 London School of Hygiene & Tropical Medicine, London, England

**Keywords:** COVID-19, Ghana, private health sector, health system

## Abstract

The private health sector is a major service provider group within the health systems of most sub-Saharan African countries. Currently, 90% of COVID-19 cases in Ghana are in urban areas where private health facilities play a major role in service delivery. Yet, there is limited understanding of the issues mediating private health sector participation in public health emergency responses. Challenges related to its involvement in Ghana´s COVID-19 response include lack of clarity on its role, sub-optimal communication channels, inadequate personal protective equipment and fragmentation of the sector. Hence, operational and regulatory actions addressing these challenges will ensure the private sector is fully prepared to contribute to the COVID-19 response and future public health emergencies, thereby strengthening Ghana’s health system.

## Commentary

The emergence of the coronavirus disease 2019 (COVID-19) has challenged health systems worldwide. It has exerted unprecedented pressure on various components of the health system including availability of essential commodities such as protective masks and maintenance of adequate health workforce and essential services. To effectively address these challenges imposed by COVID-19 on the health systems of affected countries, there is the need to mobilise all available health resources, including that in the private sector and put them to optimal use. Ghana reported its first two cases of COVID-19 on March 12th, 2020. Since then, the infection has spread and as at 5th May, 2020, a total of 2,719 cases and 18 deaths have been reported [1]. In response, the government has initiated a comprehensive cross-sectoral response with the health sector as the pivot. Within the health sector, the core response has been centered on the public sector which traditionally leads key components of outbreak response such as diagnostics, resource management, health workforce trainings and data management.

We outline the key issues related to private health sector participation that have emerged in Ghana´s COVID-19 response with the aim of informing policy formulation and implementation. These policies and interventions are expected to promote active participation of the private sector in the COVID-19 response and eventually, enhance Ghana´s health system performance. The private health sector in Ghana largely consists of privately-owned hospitals and clinics, pharmacy/chemical shops and laboratory facilities. It is a significant point of entry for health care in Ghana given its sheer numbers and contribution to service delivery. Across the country, 40.2% of health facilities are privately owned whilst the government and faith-based facilities are 53.8% and 6% respectively [2]. With regards to service delivery, the 2017 Ghana living standards survey (GLSS7) indicates that majority of health consultations (51.7%) are accessed from the private health sector [3]. Additionally, the private health sector in Ghana is largely urban-centered and concentrated in the southern part of the country; 8 out of 10 health facilities in the highly urbanized Greater Accra Region in Southern Ghana are privately owned [2].

Thus it is unsurprising that as many as 68.8% of persons in Accra, reported seeking care from private medical facilities [3]. The Ashanti and Greater Accra regions which are Ghana´s 2 most urbanised regions account for about 90% of COVID-19 infections reported in Ghana as at 5th May, 2020 [1]. There is thus considerable overlap in the geographical concentration of COVID-19 cases and private health facilities in Ghana. An effective response to the COVID-19 outbreak therefore necessarily requires comprehensive engagement of the private sector particularly in the more affected urban regions where private facilities also predominate. The private facilities within these regions especially are thus an important entry point for early identification of cases and indeed management of mild cases which may not require referral to the COVID-19 designated treatment centres. Additionally, the private sector has the potential of being hubs of innovative interventions to support the outbreak response which can be leveraged across the entire health sector.

For instance, some private facilities have reportedly responded to the outbreak by implementing e-health solutions such as a COVID-19 assessment tool and tele-consultations [4]. An additional development being led by the private sector is the collaboration between a private diagnostic company with one of Ghana´s universities to develop a rapid diagnostic test (RDT) kit for the novel coronavirus [5]. These contributions showcase the potential of the private health sector to be catalysts for innovation and development to enhance the response to COVID-19. In addition to the above strengths of the private sector to support case detection and control, it is also indispensable in efforts to obtain complete national data needed to guide key aspects of the response especially disease surveillance and planning of health services. Currently, the reporting rate of private facilities on the national routine health service database, the district health information management system (DHIMS) has often been described as incomplete [6]. This sub-optimal contribution of private health facilities to service delivery data in the country introduces significant gaps in the quality of the data guiding decision making in the outbreak.

Consequently, to enhance the private sector contribution to the COVID-19 response, it will be necessary to use the opportunity to strengthen private sector contribution to national health data. Measures such as building the capacity of the private sector personnel to use existing electronic platforms will be instrumental in improving the private sector engagement and lead to improved data quality. Additionally, the existing incentives/punitive measures for (non) reporting service delivery data need to be strengthened or enforced as may be needed. Notwithstanding the private sector´s potential to contribute meaningfully to the COVID-19 response as outlined above, it has publicly reported feeling underutilized in the ongoing outbreak response activities [7]. Additionally, the sector demanded equity in the distribution of inputs such as personal protective equipment (PPE) from the government. This request was premised on their assertion that a significant number of cases identified were first suspected in private facilities hence the need to support the sector to adequately protect its workforce [7,8]. The government subsequently responded to the concerns with an assurance to provide the sector with the needed PPEs.

These fallouts of the COVID-19 response suggest that private sector engagement (PSE) in the response is sub-optimal. The public expression of the grievances is also suggestive of in-effective communication channels between the private sector and the government/public sector. COVID-19 in Ghana therefore presents an opportunity for health planners to assess and strategize on how to achieve active private sector participation in the management of outbreaks and public health emergencies. There is an urgent need for a comprehensive rapid assessment of the human and infrastructural capacity of private health facilities in the country and for the information to be factored in the national COVID-19 response plan. The findings of the assessment will inform the support the sector needs and the opportunities to build its capacity to support the country´s response more effectively. The rapid assessment can be used to establish the nature and effectiveness of existing communication channels between the private and the public-sector as well as additional channels that can potentially promote effective interactions.

Singapore, for instance has used WhatsApp groups which include both public and private practitioners in the ongoing COVID-19 response as a result of the challenges they encountered in public-private sector interactions [9]. Additionally, as part of the assessment, there is the need to establish the extent to which the private sector is represented in standing committees such as the epidemic management and rapid response committees. The sector´s involvement in these is likely to be an enabler for improved interactions and foster trust and collaboration between the private and public sectors. Specific regulatory considerations should include the scope of government´s responsibilities to absorb the financial shocks to the private sector (especially, the for-profit group). Ultimately, appropriate legislation needs to be passed to back effective private sector participation in public health emergencies. A 2011 assessment of the private sector in Ghana´s heath delivery system reported fragmentation as a major challenge due to the wide range of players including individuals, professional associations [10] and even health entrepreneurs. The same challenge is visible in the ongoing COVID-19 response.

Currently, 2 groups, the Society of Private Medical and Dental Practitioners (SPMDP) and the Private Health Facilities Association of Ghana have been at the forefront of communicating the concerns of the sector. With the two associations and in some cases, individuals communicating these concerns, it poses a challenge in efforts to successfully engage the sector. Thus, the outbreak further highlights the need for the private health sector, despite its heterogeneity to identify and mandate a common voice that can speak on its behalf collectively. To start with, the sector can identify a task force to serve as a means of mobilizing and coordinating the sector. It will also be useful to designate a spokesperson to engage the government/public sector in the ongoing outbreak whilst it establishes more permanent structures to facilitate its interactions. This will be instrumental in efforts to effectively coordinate the sector and thereby improve the public and private sector interactions required to respond to the outbreak. The private sector has expressed concern about the rising cost of providing care driven largely by rising cost of commodities such as PPEs in the COVID-19 response.

Besides the government´s efforts to support the sector, it is imperative that additional action is taken to protect the public from being adversely affected. This requires policies and interventions that will deter private health facilities from implementing cost-cutting measures that compromise the quality of care or transfer the cost to the public. These two scenarios respectively can potentially compromise the quality of the service or increase catastrophic cost which warrants that action is taken to prevent these. Hence, the appropriate regulatory and operational provisions that ensure quality assurance including staff availability, competency and adherence to infection prevention and control measures need to be strictly enforced in the COVID-19 outbreak. Also, there should be provisions which will limit ad hoc transfer of the increasing cost of care to the public. These measures to ensure that quality and affordable care is available to all, in line with the country´s aspiration for universal health coverage is crucial to ensure that quality care remains accessible.

This will ensure continued patronage of these health facilities and therefore early detection and response to COVID-19 cases. In this commentary, we have posited that private sector engagement in the COVID-19 response in Ghana is currently sub-optimal. The sector, however, has a huge potential to help spread the COVID-19 burden especially in urban areas. Additionally, it can catalyse innovation and support attainment of complete and representative data for evidence-informed policy and actions in health care delivery including the COVID-19 response. We have also highlighted the challenges in private sector participation in the response including a lack of clarity on its role as well as government´s responsibilities towards it, ineffective communication channels, fragmented private sector and risk of compromising availability of affordable quality care. These challenges provide an opportunity to initiate policies and actions to improve the country´s response and indeed the performance of its health system.

Ultimately, we have identified the need for an appropriate policy to back private sector participation in public health emergencies such as COVID-19. Pertinent to the ongoing outbreak, we believe the sector needs to have a unified voice in its engagement with the public-sector/government. We have also suggested operational and regulatory decisions and actions to address the identified challenges. In this regard, a critical recommendation made is the need to conduct a rapid assessment of the readiness of private facilities to respond to the COVID-19 outbreak and use the findings to build the capacity of the sector to respond to public health emergencies. Collectively, these policies and interventions will ensure that the private sector´s full potential to support the COVID-19 response in Ghana is unleashed.

## Competing interests

The author declares no competing interests.
